# Do Blood-Borne Viruses Affect the Progression of Labor: A Hospital-Based Pilot Study

**DOI:** 10.7759/cureus.15631

**Published:** 2021-06-14

**Authors:** Amrita Gaurav, Dhriti Kapur, Neha Verma, Anupama Bahadur, Kavita Khoiwal, Anchal Agarwal, Om Kumari, Jaya Chaturwedi

**Affiliations:** 1 Obstetrics and Gynecology, All India Institute of Medical Sciences, Rishikesh, IND; 2 Obstetrics and Gynecology, All India Institute of Medical Sciences, New Delhi, IND

**Keywords:** seropositive, faster delivery, vigilant monitoring, obstetric care, favorable outcomes

## Abstract

Background

Blood-borne viruses form the basis of enormous research on universal precautions. A paucity of research is noted regarding labor progression in seropositive women. Women testing positive for human immunodeficiency virus (HIV)/hepatitis B surface antigen (HBsAg)/hepatitis C virus (HCV) are often denied obstetric care and referred. Their need for safe delivery conditions propelled us to undertake this study to establish whether seropositive status affects labor progression or not.

Methods

Women in early labor (<4 cm cervical dilation) testing positive for HIV/HBV/HCV and delivering vaginally during the study period at All India Institute of Medical Sciences (AIIMS), Rishikesh, India, were included as Group A (n=36). The authors recruited an equal number of women with seronegative status with comparable age, parity, admission at or before 4 cm, body mass index (BMI) characteristics as Group B. They were compared in terms of effacement at 4 cm dilatation and time from 4 cm dilatation till delivery.

Results

The authors report a significant difference (p <0.05) between time to delivery between the two groups (2 hours vs. 2.43 hours in nulligravidas and multigravidas, respectively). Thirty-two (32) of 36 cases were already 70%-80% effaced at 4 cm dilation while only 25% of controls had similar findings. The present study suggests that seropositive women progress significantly faster in labor and need vigilant monitoring. We report such findings for the first time and aim to encourage similar research worldwide.

## Introduction

Worldwide, there are approximately 1.4 million human immunodeficiency (HIV)-positive women who become pregnant each year. In India, the prevalence of hepatitis B ranges between 4.1% and 8.4% during pregnancy [1-2]. Also, women at high risk for HIV are likely to be at risk for hepatitis B virus (HBV) or hepatitis C virus (HCV), enabling co-infection with these viruses as a common event [3-5]. Guidelines on universal precautions against bloodborne viruses are regularly updated, and studies to minimize vertical transmission have often been done [6]. However, no research has yet been done to investigate the effects of the seropositive status of women on their progression of labor.

With time in the delivery room, it was noticed by our team members that seropositive women had a comparatively shorter duration of labor. The authors did not find similar studies in the literature to report evidence of the same. In India, we cannot ignore that these women are often deprived of standard intrapartum care leading to unnecessary referrals [7-8]. It thus became imperative to draw attention towards differences that might exist in their course of labor.

With this background, we planned this pilot study, in which we tested our hypothesis that the duration of labor is shorter in seropositive women than in the general population. As a secondary objective, this study also attempted to estimate the proportion of seropositive women referred to our institute. Establishing these results would help better labor monitoring and ensure avoidance of untimely referrals and enable safe, supervised childbirth.

## Materials and methods

The present study is a retrospective observational study from January 2019 to December 2019 at the Department of Obstetrics and Gynecology, All India Institute of Medical Sciences, Rishikesh, a tertiary care center in North India. Data were collected from the Medical Records Department after approval from the hospital administration. Approval of the study was obtained from the Institutional Ethics Committee of the All India Institute of Medical Sciences, Rishikesh, letter number AIIMS/IEC/20/438. Women who delivered at AIIMS Rishikesh in the study period with HIV/hepatitis B surface antigen (HBsAg)/hepatitis C virus (HCV) positive status diagnosed antenatally, who were monitored at least from 4 cm dilation (per vaginally), term, cephalic presentation, and had no other medical/obstetrical comorbidities were recruited in the study (Group A). Also, an equal number of women with HIV/HBsAg/HCV negative status and no other medical or obstetrical comorbidities, who delivered at around the same time or the next delivered woman under the supervision of the same obstetrical team in the institute, were recruited in the study (Group B). Both women were comparable in terms of age (18-30 years), parity, admission at or before 4 cm, dilation augmentation method, and body mass index (BMI) characteristics. Women presenting in the advanced stage of labor (>4 cm of cervical dilatation) and women who needed emergency or elective cesarean delivery for obstetric indication were excluded from the study. 

No regional anesthesia for pain relief was found to be administered to both groups. No other confounders were noted. None of the women required instrumental deliveries amongst both groups. Women were recruited so that they were evaluated either by the same team or teams with similar expertise. The outcome measured by the team was the cervical effacement at 4 cm dilatation and the duration of labor from 4 cm dilatation to delivery of the baby.

The seropositive cases had proven positivity by a diagnostic chemiluminescence immunoassay test at the time of admission. Per vaginal findings during labor were noted from the medical records. It was noted that they had been kept minimal and every 4 hours/on bearing down as per the protocol of the labor room. The effacement at 4 cm, along with the time taken from 4 cm dilation till delivery of the baby too, was taken from those records. As a protocol, labor monitoring was done using partograms of both groups to ensure uniformity of assessment between the two groups.

Statistical analysis

Data were collected and entered in Microsoft Excel 2010 (Microsoft Corporation, Redmond, WA). Different statistical analyses were performed using the Statistical Package for the Social Sciences (SPSS) software version 22 (IBM Corp. Armonk, NY). Data were checked for normal distribution using the Shapiro- Wilcoxon test. Effect on continuous variables was analyzed between the two groups using the Mann-Whitney U‑test. The difference of mean within groups was compared using the chi‑square test. The level of significance was kept at 5%. Data were presented as mean and standard deviation, and p<0.05 was considered statistically significant.

## Results

The time is taken from 4 cm dilatation till delivery, and the effacement at 4 cm was compared, and thus the rate of labor progression was analyzed between the two groups. Women in both groups could not be monitored from the onset of the latent phase of labor as they reached the hospital in different stages of latent labor, therefore assessment of the duration of the latent phase of labor could not be done. As it was a retrospective study, the observers had been unbiased in reporting their findings. While analyzing the reported findings, the co-investigators were blinded to whether the patient belonged to Group A or Group B.

Between January 1, 2019, to December 31, 2019, there were 45 vaginal deliveries of seropositive women. Of these, 45, mine were referred in active labor and 30 were referred in early labor. Only six cases were booked cases at our center. This pointed towards the high rate of referrals of seropositive women. More so, two of the nine women referred in active labor and had delivered in the hospital triage area itself.

In the designated study period, 36 seropositive women met the inclusion criteria and were included in the study as Group A. These women were further divided into three groups. Group A 1 comprising nulliparas had 18 women, Group A 2 comprising women with previous one normal delivery had 10 women. Group A 3 had women with previous two or more normal deliveries and included eight women. Authors included an equal number of seronegative women in Group B. Group B comprised 18 nulliparous women (B 1), 10 women with previous one normal delivery (B 2), and eight women with previous two or more normal deliveries (B 3) (Table 1).

**Table 1:** Distribution of women in Group A and Group B

Parity	GROUP A (n=36)	GROUP B (N=36)
Nullipara	A 1 (n=18)	B 1 (n=18)
Para 1	A 2 (n=10)	B 2 (n=10)
Para 2 or more	A 3 (n=8)	B 3 (n=8)

The demographic characteristics of both groups were noted and compared (Table 2).

**Table 2:** Demographic characteristics of women in Groups A and B

Parameters	Group	
	A (n = 36)	B (n = 36)
Age (Years)	20.52 yrs	21 yrs
Age***		
<20 Years	6	6
20-24 Years	28	28
25-29 Years	2	2
Occupation		
Housewife	34	33
Professional	1	2
Skilled Worker	0	1
Unskilled Worker	1	0
Education		
Illiterate	5	4
Primary	16	16
Middle	2	1
High School	6	7
Intermediate	3	2
Graduate	3	4
Postgraduate	1	2
Socio-Economic Status		
Upper	0	1
Upper middle	1	2
Lowe middle	11	9
Upper lower	16	15
Lower	8	9
Parity		
P1	18	18
P2	10	10
P3	8	8
Region		
Hilly	6	6
Plain	30	30

In Group A, 30 women were unbooked at the place of study, whereas six women were booked. A total of 24 women were booked outside the place of study. Ten out of 36 women were counseled regarding seropositive status. In Group B, 30 women were booked at the place of study, whereas two women were booked. Two women were booked outside the place of study. The clinical characteristics of women in Group A and Group B are shown in Table 3. There were no differences between the two groups regarding parity, ethnicity, co-morbidities, and BMI.

**Table 3:** Clinical characteristics of women in Groups A and B

Parameters	Group	
	A (n = 36)	B (n = 36)
BMI	22.5	22
Hemoglobin		
<10	7	7
>10	29	29
Presenting complaint		
Pain Abdomen	30	30
Leaking p/v	6	6
Obstetric Examination (Per abdomen)		
Mild contractions	20	18
Moderate contractions	16	18
Severe contractions	--	--

Progression of labor was assessed by effacement at 4 cm (Table 4), and time was taken from 4 cm dilation to delivery of the baby in the three subgroups of both groups (Table 5).

**Table 4:** Effacement in the three subgroups of Groups A and B

	GROUP A (Mean effacement as the length of the cervix in cm)	GROUP B (Mean effacement as the length of the cervix in cm)	P-VALUE
SUBGROUP 1 (n=18)	1.3±0.68,	3.09±0.44	<0.0001
SUBGROUP 2 (n= 10)	1.32±0.55	3.1±0.49	<0.0001
SUBGROUP 3 (n=8)	1.34±0.49	2.24±0.37	0.0044
Total (n=36)	1.32±0.57	2.81±0.43	<0.0001

**Table 5:** Mean duration of active labor in the three subgroups of Groups A and B

	GROUP A (mean duration in hours)	GROUP B (mean duration in hours)	P-VALUE
SUBGROUP 1 (n=18)	3.388±0.6	5.388±1.52	0.001
SUBGROUP 2 (n= 10)	2.4±0.54	3.4±0.65	0.001
SUBGROUP 3 (n=8)	2.37±0.75	3.31±0.55	0.432
Total (n=36)	2.9±0.75	4.0±1.39	0.001

None of the women in Group A had received antiviral therapy in the pre-conception period. Thirty-two of 36 women were already >70%-80% effaced at cervical dilation of 4 cm while only four of 36 women in Group B had similar findings.

In all three subgroups, Group A women took a shorter duration to deliver than Group B (Table 5). In Group A 1 (obstetric nulliparas) and Group A 2 (previous 1 normal delivery), the difference in mean duration was two hours and one hour, respectively, which was statistically significant (p-value <0.05). In Group A 3 (previous 2 normal vaginal delivery), the mean duration was 2.37 hours only, which was alarmingly short. Overall, there was a significant difference in the duration of labor between Group A and Group B (Table 5).

## Discussion

The present study points that the progress of labor in women affected with blood-borne viruses was significantly faster than in non-affected women. It also points towards better initial cervical effacement in early labor itself, giving a possible explanation for faster cervical dilation. And last but not least, the results further reiterate that a lot of women are being refused intrapartum care and are referred even if in labor.

Zhang et al. formed the foundation for new guidelines for the progression of labor and the need for cesarean delivery, which are put forth by the American College of Obstetrics and Gynecology and the Society for Maternal-Fetal Medicine [9]. According to this data, in nulliparas, the median time to progress from 4 to 5 cm was 1.3 hours, 0.8 hours to progress from 5-6 cm, and thereafter, each additional centimeter gained approximately 0.5 hrs. Also, previous studies have established that the second stage of labor lasts for a median duration of 50 minutes in nulliparas [10-11]. On the other hand, the time taken in the present study by seropositive nulliparas (Group A 1) from 4 cm till the end of the second stage of labor was a mean of 3.38 hours. This was two hours shorter than the mean duration of the seronegative group(Group B 1).

In multiparas, too, Group A women delivered in significantly shorter duration than controls or compared to the expected duration based on existing guidelines. Special attention needs to be drawn to women with previous two normal deliveries who took less than 2.3 hours. If these women are referred when in labor, it could prove catastrophic to the laboring lady, who might deliver while going to a referral center.

A possible explanation for this fast progression of labor could be attributed to prostaglandin levels. Prostaglandin E2 (PGE2), an eicosanoid generated by cyclooxygenases, has been shown to modulate inflammation and the immune system by regulating the expression/concentration of cytokine in viral infections [12]. Women infected with HIV-1 show an increase in cervical cyclooxygenase-2 (COX-2) and elevated systemic PGE2 levels [13]. Since all our cases had not received any antiviral treatment, as they were diagnosed in pregnancy itself, the chances of active viral infection and raised levels of PGE2 and COX-2 at the time of delivery are high in these women. These could contribute to faster progress, as studies suggest that cervical ripening is an inflammatory event. Practically too, for cervical ripening and improving effacement, induction with prostaglandin E2 is a tried and tested method. More so, factors such as nuclear factor (NF) kappa B, platelet-activating factor receptor (PAF-R), COX-1, and COX-2 are also reported to participate in the sequence of events leading to the final cervical ripening [14]. Raised levels of these factors in untreated seropositive women could explain the early cervical effacement and the faster progression of labor in these women. There could be other possible reasons as well, which need to be explored further.

The present study aims to draw attention to the discrimination faced by these women in terms of antenatal care and intrapartum services. In a previous study conducted to note the knowledge, practice, and attitude of providers in rural public healthcare in two states of India towards HIV-affected women, only half of the staff at community health centers (CHCs) and a quarter at primary health centers (PHCs) were confident that they could manage antenatal care for women with HIV positivity. In contrast, another quarter of PHCs mentioned that these conditions should not be managed at their centers. This reasons out the unnecessary referrals faced by these women.

The present study goes on to prove this discrimination. Thirty-nine out of 45 seropositive women were referred to our center. The fact that nine of these 39 referred women were in active labor draws immediate attention. None of these women referred had a valid indication for referral. The evidence speaks of the lack of facilities for these laboring women at private centers and PHCs [7-8]. If the time to delivery is over-estimated (or considered to be the same as that of the general population), and the current study findings are ignored, these women stand at a greater risk of delivering on the way to the referral center or at home or in the triage area of the referral center. This not only puts the laboring woman and her baby at risk but also exposes the medical professional to seropositive body fluids due to the mere lack of time on presentation.

Our study thus aims to highlight the faster progress of labor in seropositive women and the need for better monitoring and more vigilant care. It also aims to warn the health care workers regarding avoidance of unnecessary referrals and emphasizes not overestimating the time in hand when referring seropositive women. This study presses on the need for adequate antenatal counseling and timely referrals of the antenatal women in case the center is not willing or adequately trained/equipped to conduct seropositive deliveries.

We acknowledge several limitations of the study. The study was not population-based and had a limited sample size. It was a single institute study. Owing to the retrospective nature of this study, we could not control for inter-observer variations, especially in per vaginal findings. We had to limit ourselves to the information previously obtained. We could not get viral loads or prostaglandin levels done to find a possible cause or justify the hypothesized PGE2 theory as an explanation for the faster progress of labor. Nonetheless, this study aims to spark further research on these grounds. As this was a retrospective study, it was not practically possible for the investigators to have known/noted the exact time of full dilation. Hence, the endpoint of the outcome for this retrospective study was chosen to be the end of the second stage (delivery of the baby). However, it is accepted by the investigators that a more specific endpoint in such studies could be when the woman is fully dilated. If this were known, it would ensure a more specific measurement of the first stage of labor exclusively.

Interobserver bias was involved in the study, as findings were not by the same individual, rather by the same team with similar capabilities. However, it was minimized, as the team of clinicians was the same or similar for both the groups. Comparability was ensured between the two groups by the principal investigator.

Multiple similar multicentric prospective trials need to be done to ensure validation of the findings of this study. This study aims to ignite multiple more studies to find the causal factors for the hypothesis described.

## Conclusions

The present study concludes that women with blood-borne viral infection have an earlier effacement and progress faster in labor. While the association does not necessarily imply causation, there are good physiological grounds for expecting some causal relationship, and the findings should not be ignored. Further studies are needed to validate our results, establish causality, and prevent the discrimination faced by these women.
